# The role of NFκB in spheroid formation of human breast cancer cells cultured on the Random Positioning Machine

**DOI:** 10.1038/s41598-017-18556-8

**Published:** 2018-01-17

**Authors:** Sascha Kopp, Jayashree Sahana, Tawhidul Islam, Asbjørn Graver Petersen, Johann Bauer, Thomas J. Corydon, Herbert Schulz, Kathrin Saar, Norbert Huebner, Lasse Slumstrup, Stefan Riwaldt, Markus Wehland, Manfred Infanger, Ronald Luetzenberg, Daniela Grimm

**Affiliations:** 1 Clinic for Plastic, Aesthetic and Hand Surgery, Otto-von-Guericke-University Magdeburg, D-39120 Magdeburg, Germany; 2 Department of Biomedicine, Aarhus University, Wilhelm Meyers Allé 4, DK-8000 Aarhus C, Denmark; 3 Max-Planck Institute of Biochemistry, D-82152 Martinsried, Germany; 4 Department of Ophthalmology, Aarhus University Hospital, DK-8000 Aarhus C, Denmark; 5 Cologne Center for Genomics, University of Cologne, D-50931 Cologne, Germany; 6 Max-Delbrück-Center for Molecular Medicine, D-13092 Berlin-Buch, Germany

## Abstract

Human MCF-7 breast cancer cells were exposed to a Random Positioning Machine (RPM). After 24 hours (h) the cells grew either adherently within a monolayer (AD) or within multicellular spheroids (MCS). AD and MCS populations were separately harvested, their cellular differences were determined performing qPCR on genes, which were differently expressed in AD and MCS cells. Gene array technology was applied to detect RPM-sensitive genes in MCF-7 cells after 24 h. Furthermore, the capability to form multicellular spheroids *in vitro* was compared with the intracellular distribution of NF-kappaB (NFκB) p65. NFκB was equally distributed in static control cells, but predominantly localized in the cytoplasm in AD cells and nucleus in MCS cells exposed to the RPM. Gene array analyses revealed a more than 2-fold change of only 23 genes including some whose products are affected by oxygen levels or regulate glycolysis. Significant upregulations of the mRNAs of enzymes degrading heme, of *ANXA1*, *ANXA*2, *CTGF*, *CAV2* and *ICAM1*, as well as of *FAS*, *Casp8*, *BAX*, *p5*3, *CYC1* and *PARP1* were observed in MCS cells as compared with 1*g*-control and AD cells. An interaction analysis of 47 investigated genes suggested that *HMOX-1* and NFκB variants are activated, when multicellular spheroids are formed.

## Introduction

Exposing cells to devices like the Random Positioning Machine (RPM) triggers them to change their growth behavior together with a number of cellular characteristics^1,2^. This phenomenon has been observed for several types of human cells including thyroid cells, chondrocytes, endothelial cells, human breast cancer MDA-MB-231 cells and others^3–9^. We recently demonstrated that also human Michigan Cancer Foundation-7 (MCF-7) breast cancer cells form three-dimensional (3D) aggregates. Incubated on a RPM, a part of the cells switches from a two-dimensional (2D) growth within a monolayer to a 3D growth within multicellular spheroids (MCS), the other one remains adherent and continues growing within a monolayer (AD)^10^. The occurrence of MCS begins within 24 h on the RPM^10^. After a five-day RPM-exposure, spheroids were floating in the culture supernatant. At that time, the cells of the MCS have changed their gene expression pattern. Among other mRNAs, vascular endothelial growth factor-A (*VEGFA*), vascular endothelial growth factor receptor 2 (*FLK1*), caspase-9 (*Casp9*), caspase-3 (*Casp3*), and protein kinase C alpha (*PRKCA*) mRNAs were downregulated in five-day MCS-samples indicating their involvement in 3D aggregation.

Isochronally with 3D aggregation, microgravity-induced apoptosis was detected in breast cancer cells^6^ like it has been observed in other types of cells^11–13^. In addition, FTC-133 thyroid cancer cells exposed to the RPM for 24 h formed spheroids and exhibited simultaneously enhanced levels of apoptosis and of NFκB proteins as compared with 1*g*-control cells^14^. NFκB incorporates a variety of transcriptional regulatory functions and is known to be of great importance in apoptosis^15^. It is inactivated by binding to IκB (inhibitor of NFκB). However, degradation of IκB can result in a translocation of NFκB into the nucleus, where it can activate the transcription of anti-apoptotic genes^16^. In a recent deep proteome analysis the translocation inhibitor IκBKB showed up in AD cells after culturing FTC-133 cells on the RPM, but could not be detected in MCS cells of the same culture flask^17,18^. These observations created the idea that a link between spheroid formation, initiation of apoptosis and NFκB expression may exist^14^. In addition, Becker-Weimann *et al*. postulated a link between NFκB expression and 3D organization of human breast cancer cells^19^.

Therefore, the principal aim of this paper was to investigate the early phases of RPM-exposure (24 h) of MCF-7 breast cancer cells and to test whether there is a link between enhancement of apoptosis, changes in NFκB expression and spheroid formation. In a first approach, we exposed MCF-7 breast cancer cells for 24 h to the RPM. Afterwards, we analyzed the intracellular distribution and expression of NFκB by means of gene array analyses as well as quantitative (q)PCR focusing on genes involved in apoptosis and cell adhesion signaling and known to play a role in spheroid formation of human thyroid cells^14^. Furthermore, morphological and molecular biological results were compared. These experiments should increase the knowledge about mechanisms of the self-reliant formation of tissue-comparable cell-aggregates. Finding molecules in various cell types, which mediate a microgravity-dependent cell organization in equal ways, may indicate new targets to improve tissue engineering and cancer treatment. In a further step, we investigated the impact of the poly ADP ribose polymerase (PARP) inhibitor olaparib, the effect of dexamethasone (DEX) and the phosphodiesterase-4 (PDE-4) inhibitor rolipram on spheroid formation.

## Results

**Figure 1:**
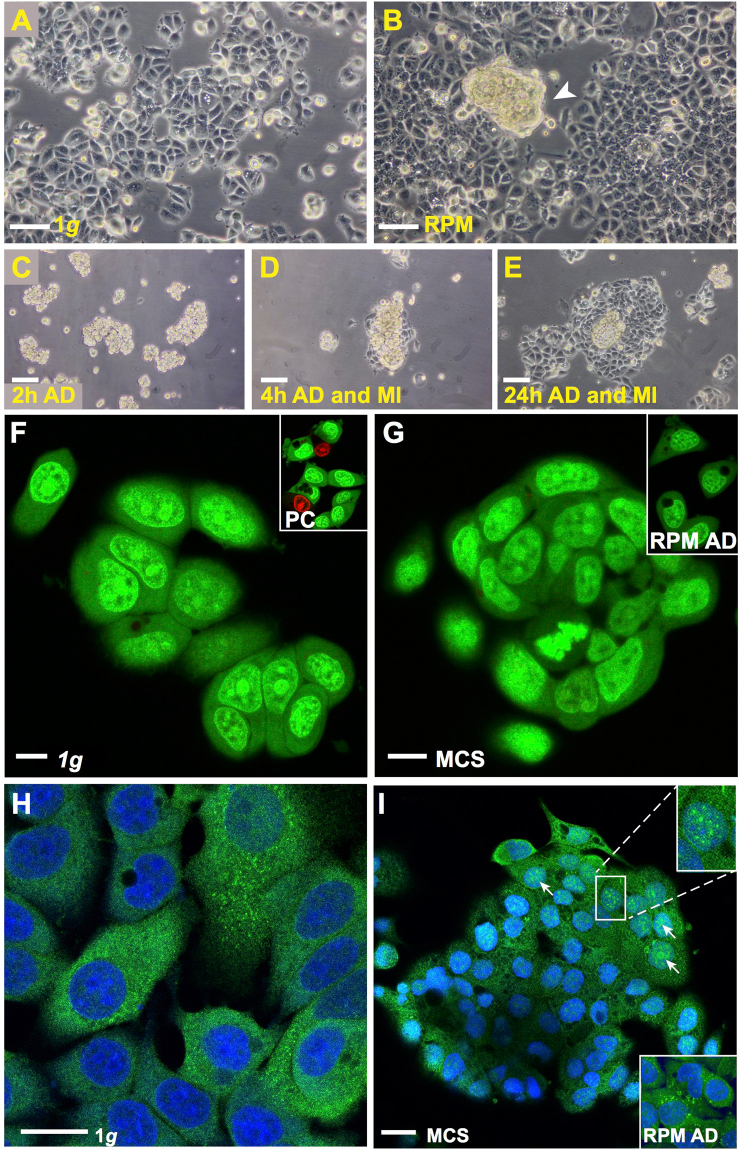
(**A**–**E**) Phase contrast microscopy: (**A**) Native MCF-7 cells cultured for 24 h under 1*g*-conditions. (**B**) RPM-exposed sample showing two phenotypes (adherently growing MCF-7 cells and MCS). A representative example of a MCS is indicated by the white arrowhead. (**C**) Adhesion of MCS to the bottom of a slide flask after 2 h. (**D**) Migration of MCF-7 cells out of the MCS after 4 h and (**E**) Migration of MCF-7 cells out of the MCS after 24 h. (**F**–**I**) Confocal laser scanning microscopy: (**F**,**G**) Acridine orange/ethidium bromide staining revealed a green fluorescence in all cells after 24 h which indicates viability. (**F**) 1*g*-conditions (insert: positive control (PC) of acridine orange/ethidium bromide assay after approximately 5 min incubation). (**G**) viable cells in the MCS (insert: viable RPM-AD cells). (**H**) NFκB immunofluorescence staining of 1*g*-control cells: NFκB is predominantly detectable in the cytoplasm and I: RPM-exposed samples: nuclear (white arrows) and cytoplasmatic NFκB in the MCS. Upper insert: magnification of indicated area. Lower insert: NFκB detection in RPM AD cells, same magnification as in I. Nuclei are counterstained with DAPI. Scale bars in (**A**–**E**) 100 µm; scale bars in (**F**,**G**) 10 µm; scale bars in (**H** and **I**): 20 µm. 5 samples of each condition were examined separately.

After MCF-7 human breast adenocarcinoma cells had been cultured on the RPM for 24 h, we detected two different phenotypes: Cells growing adherently within a 2D monolayer (AD) and cells growing in form of 3D aggregates exhibiting no glandular structures after this short-term exposure. The MCS had various sizes (max. 300 µm) and were floating in the supernatant (Fig. 1B). In the corresponding controls incubated under normal 1*g-*conditions only cells growing in 2D monolayers could be seen (Fig. 1A). The cells growing three-dimensionally in form of MCS were viable. This was demonstrated by testing the adhesion of the MCS (Fig. 1C) on slide flasks and by the migration behavior of the cells clearly seen at 4 h of incubation (Fig. 1D). After 24 h a large number of cells are migrating out of the MCS (Fig. 1E). In addition, acridine orange/ethidium bromide staining showed that MCS cells were viable and impermeable to the dye (Fig. 1G; green fluorescence) like the RPM AD cells (Fig. 1G; insert) and the 1*g*-control MCF-7 cells (Fig. 1F). In case of dead or necrotic cells, the cells would have taken up the dye and shown a red fluorescence. This is demonstrated in the insert of Fig. 1F, where red cells are visible when stained living cells are kept without medium under the microscope for another 5 min and photographed afterwards.

### Effect of RPM-exposure on NFκB

In order to see, whether the NFκB-p65 location and content of MCF-7 cells is like that of FTC-133 follicular thyroid cancer cells^14^ involved in the cellular processes taking place during the first 24 h of RPM-exposure, we performed a NFκB-p65 immunofluorescence staining and Western blot analyses. The antibodies used are listed in Table 1. The Fig. 1H and I reveal MCF-7 cells stained for NFκB-p65. It can be seen that NFκB-p65 is equally distributed in the cytoplasm of cells cultured under static 1*g*-conditions (Fig. 1H). After a 24-hour RPM-exposure NFκB-p65 protein appeared in the nucleus of MCS cells (Fig. 1I; upper insert). Fluorescence brightness shown in the upper and lower inserts of Fig. 1I suggested more NFκB-p65 in the nucleus and cytoplasm of MCS cells than in the cytoplasm of adherent cells exposed to the RPM.

**Table 1:** Antibodies applied for Western blot Analysis.

Antibody	Dilution	Company	Molecular weight	Catalog Number
p-NFκB p50	1/1000	Thermo Fisher	50 kDa	#710460
p-NFκB p52	1/1000	Thermo Fisher	110 kDa	#PA5-17385
Anti-NFκB p105/p50	1/1000	Abcam	50, 100 kDa	#ab32360
Anti-p-NFκB p65	1/1000	Abcam	70 kDa	#ab86299
Anti-IκBα	1/1000	Cell-Signaling	39 kDa	#9242
Anti-p-IκBα	1/1000	Cell-Signaling	40 kDa	#2859
Anti-NFκBp65	1/1000	Cell-Signaling	65 kDa	#C22B4
Anti-Cofilin	1/1000	Abcam	19 kDa	#ab124979

**Figure 2:**
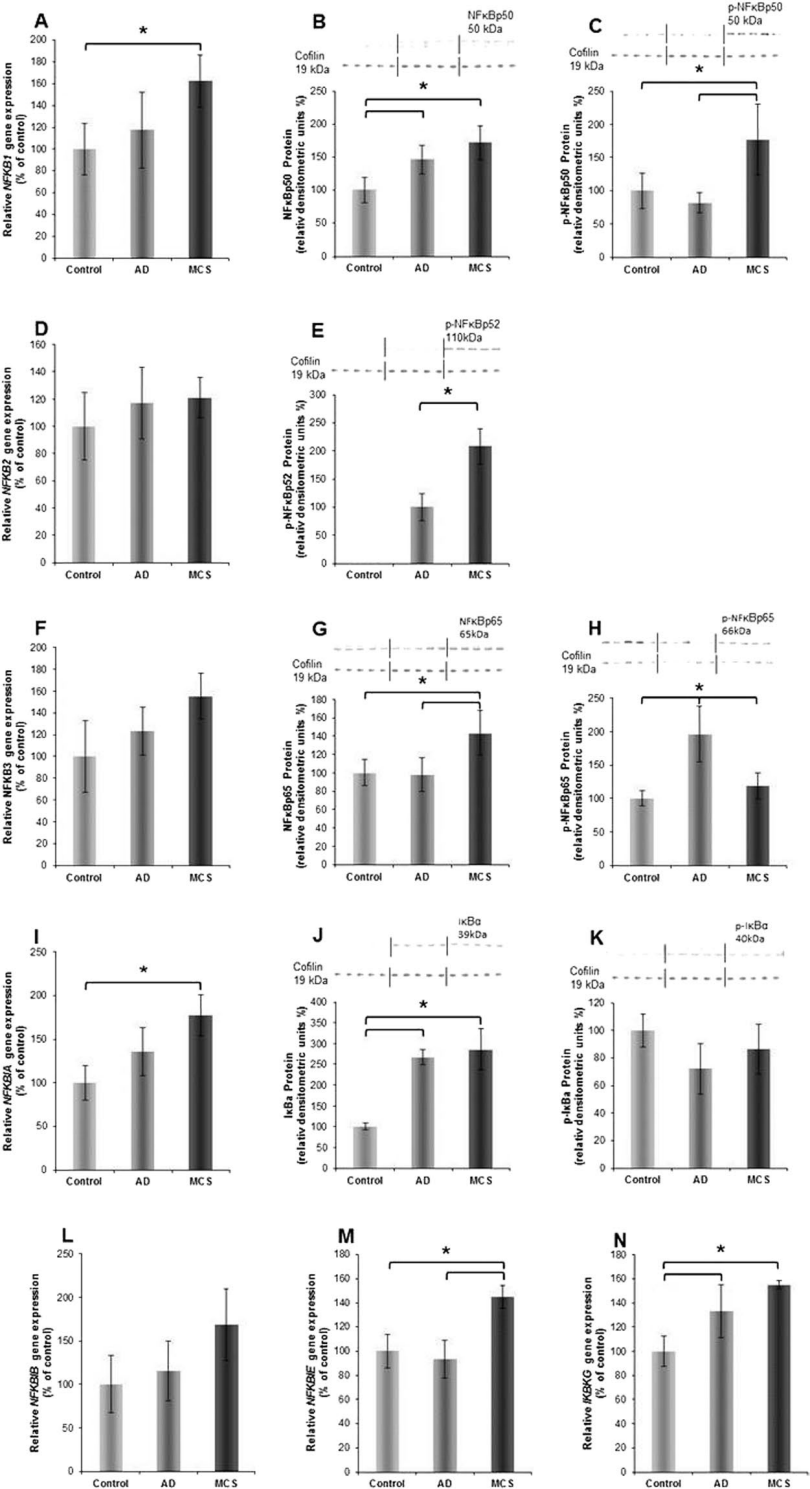
(**A**) *NFKB1* gene expression; (**B**) NFkBp50 Western blot analysis; (**C**) phosphorylated (p)-NFκBp50 Western blot analysis; (**D**) *NFKB2* gene expression; (**E**) p-NFκBp52 Western blot analysis; (**F**) *NFKB3* gene expression; (**G**) NFκBp65 Western blot analysis; (**H**) p-NFkBp65 Western blot analysis; (**I**) *NFKBIA* gene expression; (**J**) IκBα Western blot analysis; (**K**) p- IκBβ Western blot analysis; (**L**) *NFKBIB* gene expression; (**M**) *NFKBIE* gene expression; (**N**) *IKBKG* gene expression. The position (arrow) and molecular size (in kD) of the investigated proteins are indicated on each of the Western blot membrane images. Cofilin 1 was used as loading control. The vertical lines indicate group separation giving n = 5 per group.

NFκB proteins comprise different variants including NFκB-p50, -p52 and -p65. They are encoded by the gene loci *NFKB1*, *2* and *3*. The various proteins form dimeric transcription factors that regulate the expression of genes influencing a broad range of biological processes^20–22^. NFκB proteins are bound and inhibited by IκB proteins. Both, effectors and inhibitors may be activated by external signals, which trigger expression, phosphorylation and dimerization of various components as well as their translocation from the cytoplasm to nucleus, where it binds to specific DNA sequences (response elements). In order to investigate whether the differences in localization and amount of NFκB-p65 observed in Fig. 1H and I are only due to a re-distribution or are also due to a new synthesis of NFκB-p65 proteins, we performed qPCR of the *NFKB1*, *NFKB2*, *NFKB3*, *NFKBIA*, *NFKBIB*, *NFKBIE and NFKBG* genes (Fig. 2). Figure 2D,F,L indicate a tendency of a non-significant upregulation of *NFKB2*, *NFKB3*, *NFKBIB* genes under simulated microgravity (s-µ*g*) in AD cells and especially in MCS, while Fig. 2A,I,M,N show a significant upregulation of *NFKB1*, *NFKBIA*, *NFKBIE and NFKBG* genes in MCS cells as compared to control cells. In AD cells only the *IKBKG* gene is significantly enhanced in comparison to the control cells. In general, the Western blot analyses performed on the same group as the qPCR, point in a similar direction as the corresponding gene expression pattern along with the corresponding un-phosphorylated proteins (Fig. 2B,G,J). Interestingly, the phosphorylated (p−) variants of the proteins are enhanced mainly in RPM-exposed cells (Fig. 2C,E,H). Hence, a significant phosphorylation of NFκB proteins during MCS formation on the RPM may be considered.

### Genes of factors known to be involved in spheroid formation

**Figure 3:**
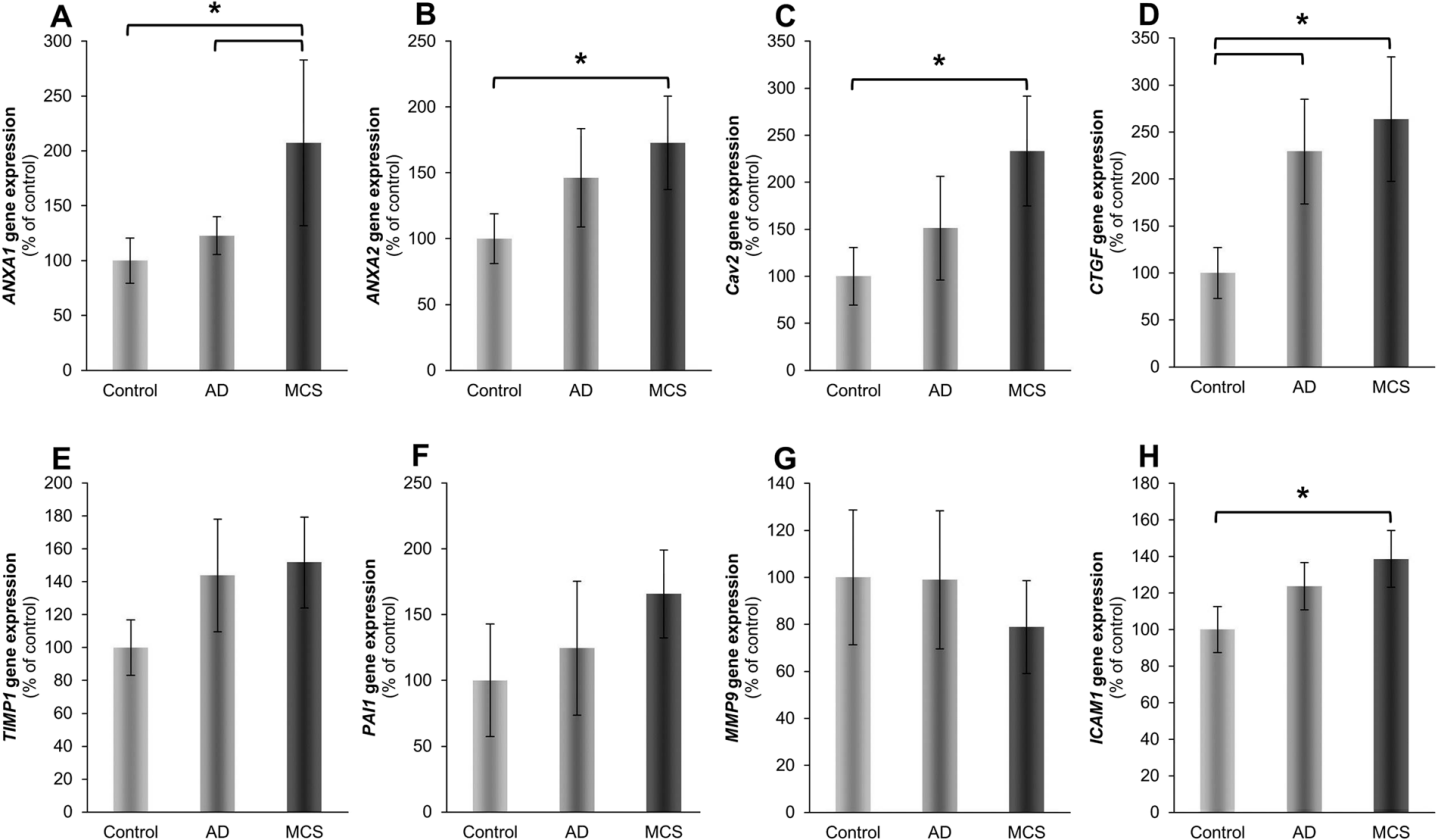
Expression of genes putatively involved in MCS-formation. (**A**) Annexin A1 (*ANXA1*), (**B**) Annexin A2 (*ANXA2*), (**C**) Caveolin-2 (*Cav2*), (**D**) Connective tissue growth factor (*CTGF*), (**E**) Metalloproteinase inhibitor 1 (*TIMP1*), (**F**) Plasminogen activator inhibitor 1 (*PAI1*), (**G**) Matrix metalloproteinase 9 (*MMP9*), (**H**) Intercellular adhesion molecule 1 (*ICAM1*), were analysed after 24 h of RPM-exposure. All values are given as mean ± standard deviation. *p < 0.05 vs. corresponding 1*g*-control. AD: adherent cells, MCS: multicellular spheroids, after RPM-exposure. Number of replicates is 4.

Because the growth behavior, *NFKB3* gene expression and NFκB p65 protein accumulation were similar in FTC-133 and MCF-7 cells, we investigated the expression of other genes, which are assumed to play a role in the formation of 3D aggregates of human cancer cells^14,23^. The primers used are listed in Table 2. The gene expression status was studied after 24 h by qPCR in 1*g*-control cells, AD and MCS cells comparatively. These qPCR experiments revealed: The gene expression of annexin A1 (*ANXA1*) and annexin A2 (*ANXA2*) were significantly upregulated in MCS compared to 1*g*-control cells, while their expression was unregulated in AD samples (Fig. 3A,B). In addition, caveolin-2 (*CAV2*) and intercellular adhesion molecule 1 (*ICAM1*) mRNAs were both elevated in MCS. The expression was significantly upregulated in MCS compared to the corresponding 1*g*-controls (Fig. 3C,H). In contrast to *CAV2*, the connective tissue growth factor (*CTGF*) gene expression was significantly upregulated in AD as well as in MCS samples (Fig. 3D).

**Table 2:** Primers used for quantitative real-time PCR

Factor	Primer name	Sequence 5′ - 3′
*18 S*	*18S-F*	GGAGCCTGCGGCTTAATTT
	*18S-R*	CAACTAAGAACGGCCATGCA
Annexin A1; *ANXA1*	*ANXA1-F*	GCCAAAGACATAACCTCAGACACAT
	*ANXA1-R*	GAATCAGCCAAGTCTTCATTCACA
Annexin A2; *ANXA2*	*ANXA2-F*	GGTACAAGAGTTACAGCCCTTATGACA
	*ANXA2-R*	CATGGAGTCATACAGCCGATCA
Apoptosis Regulator BAX; *BAX*	*BAX-F*	GTCAGCTGCCACTCGGAAA
	*BAX-R*	AGTAACATGGAGCTGCAGAGGAT
Apoptosis Regulator BCL-2; *BCL2*	*BCL2-F*	TCAGAGACAGCCAGGAGAAATCA
	*BCL2-R*	CCTGTGGATGACTGAGTACCTGAA
Caspase 3; *CASP3*	*Casp3-F*	CTCCAACATCGACTGTGAGAAGTT
	*Casp3-R*	GCGCCAGCTCCAGCAA
Caspase 8; *CASP8*	*Casp8-F*	TGCAAAAGCACGGGAGAAAG
	*Casp8-R*	CTCTTCAAAGGTCGTGGTCAAAG
Caspase 9; *CASP9*	*Casp9-F*	CTCCAACATCGACTGTGAGAAGTT
	*Casp9-R*	GCGCCAGCTCCAGCAA
Caveolin 2; *CAV2*	*Cav2-F*	GATCCCCACCGGCTCAAC
	*Cav2-R*	CACCGGCTCTGCGATCA
Connective Tissue Growth Factor; *CTGF*	*CTGF-F*	ACAAGGGCCTCTTCTGTGACTT
	*CTGF-R*	GGTACACCGTACCACCGAAGAT
Cytochrome C; *CYC*	*Cyc-F*	CACTGCGGGAAGGTCTCTAC
	*Cyc-R*	GGGGTGCCATCGTCAAACTC
NF-kappa-B transcription complex P105/P50; *NFKB1*	*NFkB1-F*	CTTAGGAGGGAGAGCCCAC
	*NFkB1-R*	TGAAACATTTGTTCAGGCCTTC
NF-kappa-B transcription complex P100/P52; *NFKB2*	*NFkB2-F*	GTACAAAGATACGCGGACCC
	*NFkB2-R*	CCAGACCTGGGTTGTAGCA
NF-kappa-B transcription complex P65	*NFkB-F*	CGCTTCTTCACACACTGGATTC
	*NFkB-R*	ACTGCCGGGATGGCTTCT
NF-kappa-B essential modulator (NEMO); *IKBKG*	*IkBKG-F*	AACTGGGACTTTCTCGGAGC
	*IkBKG-R*	GGCAAGGGCTGTCAGCAG
NF-kappa-B inhibitor alpha; *NFKBIA*	*NFkBIa-F*	AATGCTCAGGAGCCCTGTAAT
	*NFkBIa-R*	CTGTTGACATCAGCCCCACA
NF-kappa-B inhibitor beta; *NFKBIB*	*NFkBIb-F*	CCCGGAGGACCTGGGTT
	*NFkBIb-R*	GCAGTGCCGTGTCCCC
NF-kappa-B inhibitor epsilon; *NFKBIE*	*NFkBIe-F*	TGGGCATCTCATCCACTCTG
	*NFkBIe-R*	ACAAGGGATTCCTCAGTCAGGT
Tumor necrosis factor receptor superfamily member 6 (Fas); *FAS*	*CD95-F*	AGTCTGGTTCATCCCCATTGAC
	*CD95-R*	AGGGATTGGAATTGAGGAAGACT
Intercellular adhesion molecule 1; *ICAM1*	*ICAM1-F*	CGGCTGACGTGTGCAGTAAT
	*ICAM1-R*	CTTCTGAGACCTCTGGCTTCGT
Matrix metalloproteinase-9; *MMP9*	*MMP9-F*	CCTGGAGACCTGAGAACCAATC
	*MMP9-R*	TTCGACTCTCCACGCATCTCT
Cellular tumor antigen p53; *p53*	*p53-F*	CCTGGATTGGCCAGACTGC
	*p53-R*	TTTTCAGGAAGTAGTTTCCATAGGT
Plasminogen activator inhibitor 1; *PAI1*	*PAI1-F*	AGGCTGACTTCACGAGTCTTTCA
	*PAI1-R*	CACTCTCGTTCACCTCGATCTTC
Poly [ADP-ribose] polymerase 1; *PARP1*	*PARP1-F*	CGAGTCGAGTACGCCAAGAG
	*PARP1-R*	CATCAAACATGGGCGACTGC
Metalloproteinase inhibitor 1; *TIMP1*	*TIMP1-F*	GCCATCGCCGCAGATC
	*TIMP1-R*	GCTATCAGCCACAGCAACAACA

All sequences are given in the 5′–3′ direction.

Both, tissue inhibitor of metalloproteinases 1 (*TIMP1*) and plasminogen activator inhibitor 1 (*PAI1*) mRNAs were not significantly regulated, however, a tendency of an upregulation in AD and MCS was visible (Fig. 3E,F). Both factors inhibit metalloproteinases including metalloproteinase 9 (MMP9), whose mRNA was not significantly regulated (Fig. 3G).

**Figure 4:**
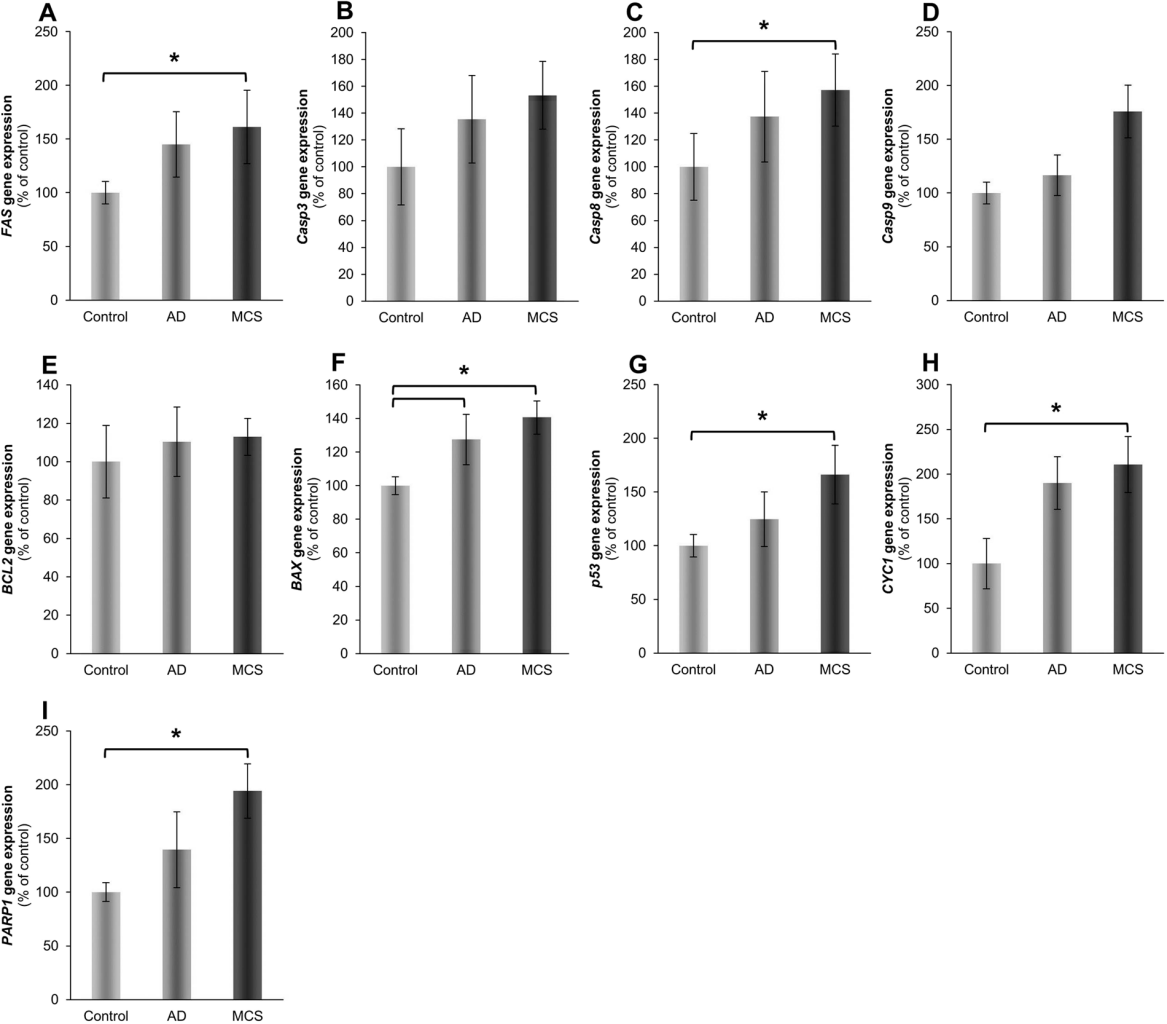
Gene expression of genes whose products are associated with apoptosis pathway. (**A**) Fas, (**B**) Caspase-3 (*Casp3*), (**C**) Caspase-8 (*Casp8*), (**D**) Caspase-9 (*Casp9*), (**E**) Apoptosis regulator Bcl-2 (*BCL2*), (**F**) Apoptosis regulator BAX (*BAX*), (**G**) Cellular tumor antigen p53 (*p53*), (**H**) Cytochrome C 1 (*Cyc1*), (**I**) Poly ADP-ribose polymerase (*PARP1*) were analyzed after 24 h of RPM-exposure. All values are given as mean ± standard deviation. *p < 0.05 vs. corresponding 1*g*-control. AD: adherent cells, MCS: multicellular spheroids, after RPM-exposure. Number of replicates is 4.

Additional genes involved in intrinsic and extrinsic pathways of programmed cell death, were selected, because apoptosis was found repeatedly to accompany cell response to removing gravity^1,5,11,14^. *Casp3* and *Casp9* mRNAs were not significantly changed in AD and MCS compared to their corresponding controls (Fig. 4B,D). However, only MCS versus control showed a significant upregulation of caspase-8 (*Casp8*) (Fig. 4C). Furthermore, the cellular tumor antigen p53 (*p53*) gene expression was significantly enhanced in MCS versus control cells (Fig. 4G). The cytochrome c1 (*CYC1*), poly(ADP-ribose)-polymerase 1 (*PARP1*) (Fig. 4H,I) and tumor necrosis factor receptor superfamily member (*FAS*) mRNAs (Fig. 4A) were upregulated in MCS samples compared to the control group. In addition, the apoptosis regulator Bcl-2 (*BCL2*) mRNA remained unregulated in AD and MCS cells (Fig. 4E). In contrast, the apoptosis regulator BAX (*BAX*) gene expression was significantly upregulated in AD and MCS samples compared to the control cells (Fig. 4F). However, many of them exhibited significant regulations in at least one of the three possible permutations of gene expression comparisons between our experimental groups.

### Microarray analysis

**Figure 5:**
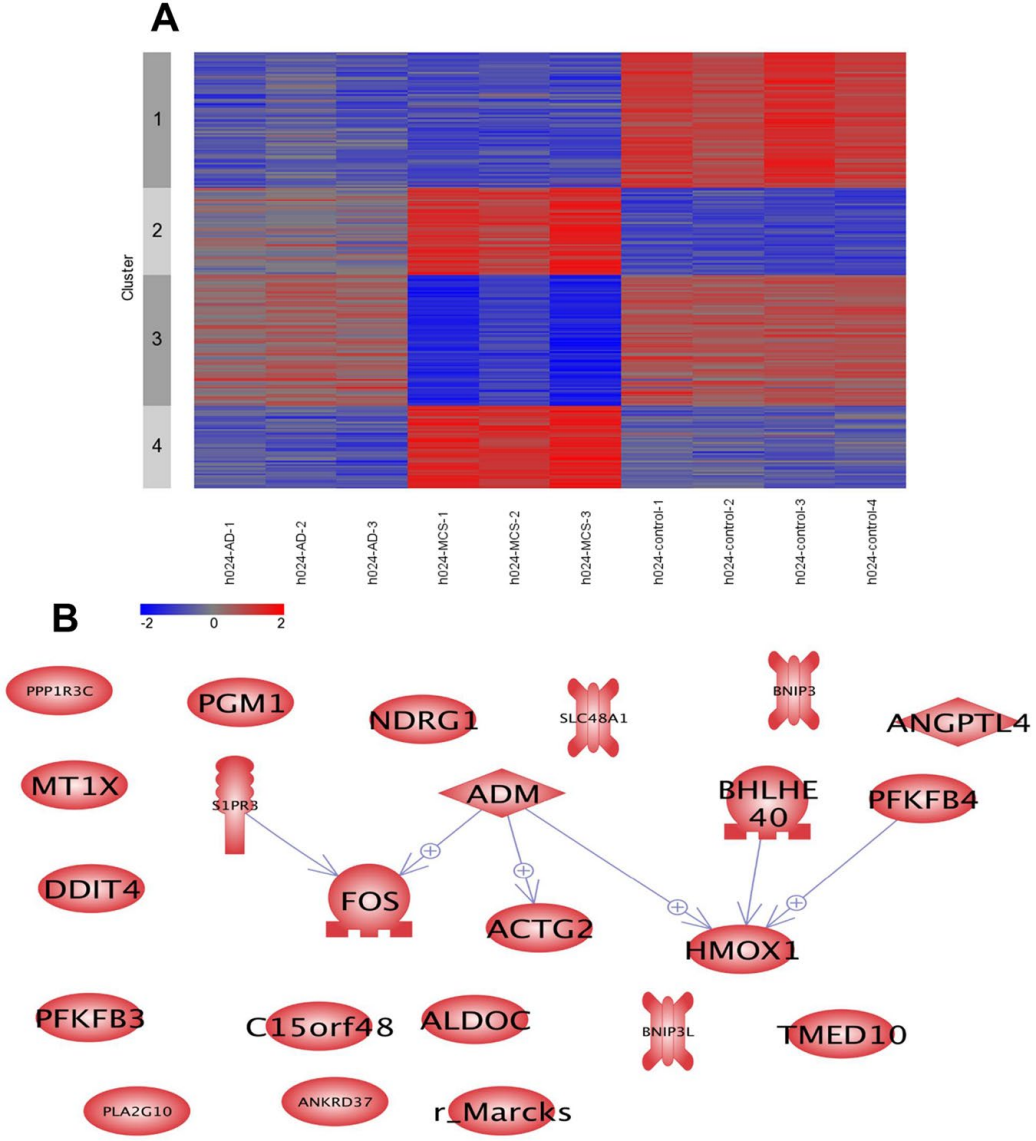
(**A**) K-mean clustering of significant expression differences in the microarray experiment (5% FDR, N = 331 probes). Using k = 4 the first cluster comprises 103 probes downregulated in MCS and AD. The second and forth cluster comprise 66 and 63 probes upregulated in MCS. Cluster 2 genes have an intermediate expression in AD. The 99 probes in cluster three are downregulated in MCS. (**B**) Pathway studio analysis of genes detected by the gene array analysis: The arrows indicate interaction. HMOX1 is most net-worked.

In order to detect further genes, which change their expression activity, during the first 24 h of culturing MCF-7 cells on the RPM, we performed microarray analyses on 1*g*-control, AD and MCS cells (Fig. 5A). The microarray analysis (Table 3 and Supplemental Table 1) did not show a significant microgravity-dependent change in *NFκB-p65* expression. Moreover, it revealed a rather stable mRNA expression pattern. In total 319 genes (331 probes, 330 probes annotated to 319 genes) transcripts were significantly differentially expressed (5% false discovery rate (FDR) in Analysis of variance (ANOVA)). In the pairwise comparison of control cells, AD and MCS, the expression of 140 significantly differentially expressed genes and open reading frames was changed 1.4-fold (Supplemental Table 1). However, a two- or more-fold change of the mRNA was merely found in 23 genes (Table 3). As a two- or more-fold change is usually considered to indicate biological relevance, we studied these genes in more detail.

**Table 3:** Genes changed two- or more-fold after a 24 h-RPM-exposure.

Gene symbol	Fold change AD vs. controls	Fold change MCS vs. controls	Oxygen
*ADM*	−3.97341	−3.71790	sensitive
*ALDOC*	−2.72112	−2.93859	sensitive
*ANGPTL4*	−2.82266	−2.52424	sensitive
*ANKRD37*	−3.30216	−3.47036	sensitive
*BHLHE40*	−2.63050	−2.12209	
*BNIP3*	−2.06507	−1.99325	sensitive
*BNIP3L*	−2.10573	−2.65328	sensitive
*C15ORF48*	−1.28973	−2.14925	
*DDIT4*	−2.36264	−2.80513	sensitive
*FOS*	−2.23903	−1.62010	
*MARCKS*	−1.23023	−2.21407	
*MT1X*	−2.30906	−2.49814	
*NDRG1*	−2.95903	−2.78486	sensitive
*PFKFB3*	−2.10202	−3.13231	sensitive
*PFKFB4*	−3.20572	−2.84829	sensitive
*PGM1*	−1.77618	−2.05219	
*PLA2G10*	−1.17541	−2.21319	
*PPP1R3C*	−1.72085	−2.07604	
*TMED10*	−1.13444	−2.60338	
*SLC48A1*	1.31299	2.07465	
*S1PR3*	1.46902	2.07908	
*ACTG2*	1.67742	2.17152	
*HMOX1*	1.23716	2.78409	

19 of the genes were downregulated more than two-fold and 4 genes were upregulated at least two-fold (Table 3). 10 of the down-regulated genes code for proteins linked to oxygen levels or hypoxia^24–30^. The degree of downregulation of the expression of these genes was very similar in AD and MCS cells. But myristoylated alanine-rich C-kinase substrate (*MARCKS*), which codes for an actin interacting protein, is more significantly (5% FDR ANOVA) downregulated in MCS than in AD cells^31^. A more profound difference between AD and MCS cells was seen, when the upregulated genes were analyzed, which code for the cytoskeletal protein gamma-enteric smooth muscle actin (ACTG) and additional three proteins suppressing apoptosis and regulating the concentration of heme, which influences apoptosis^32–34^. All 4 genes indicated were significantly upregulated (>2 fold) only in MCS cells (Table 3).

**Figure 6:**
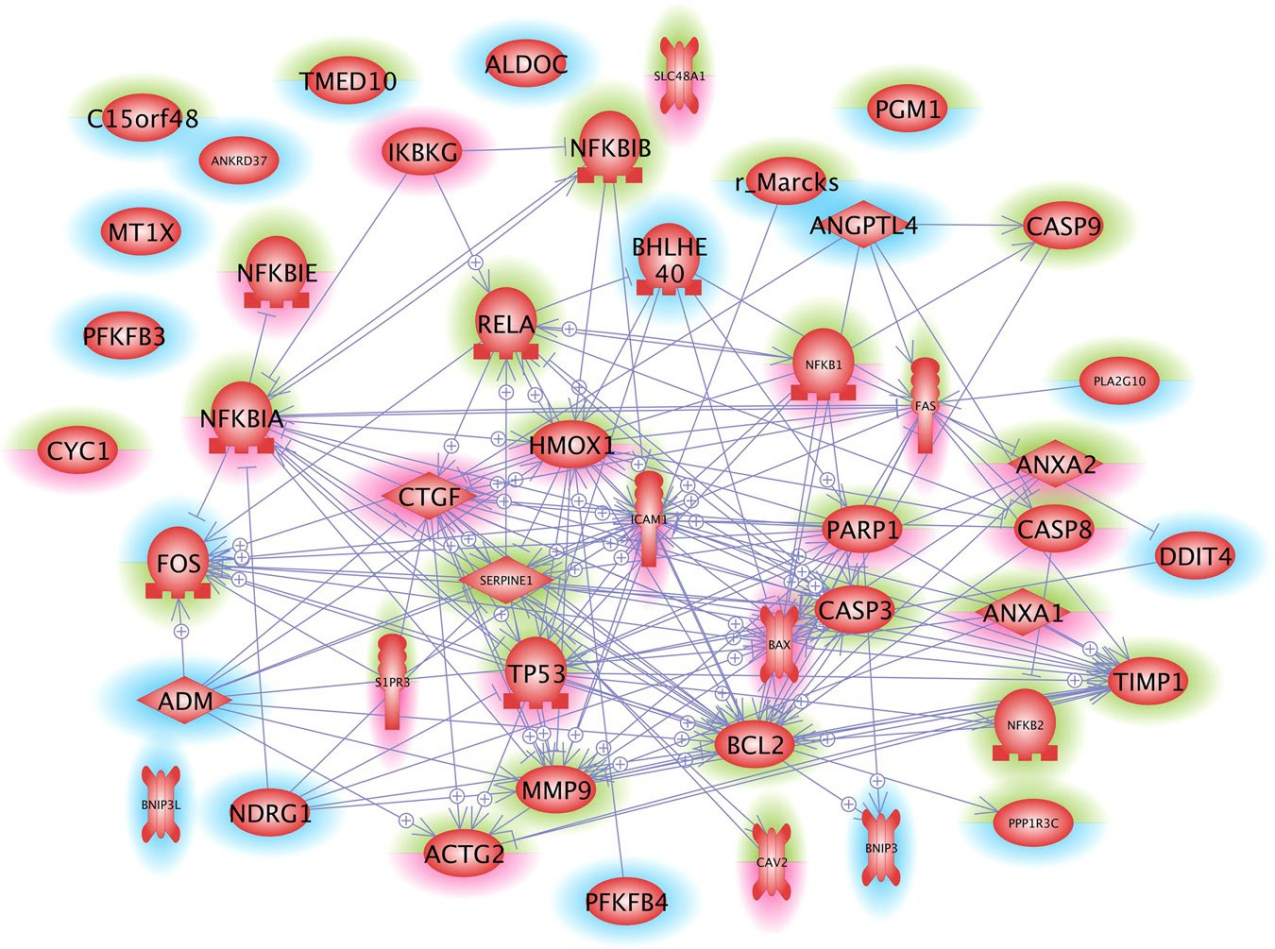
Pathway studio analysis of genes analyzed in the study by the gene array analysis and qPCR. Arrows indicate influence. Rims around the icon indicate up- (red) and down-regulation (blue). Green means un-regulated. The lower half of a rim indicates regulation in MCS, the upper half indicates regulation in the AD cells.

**Figure 7:**
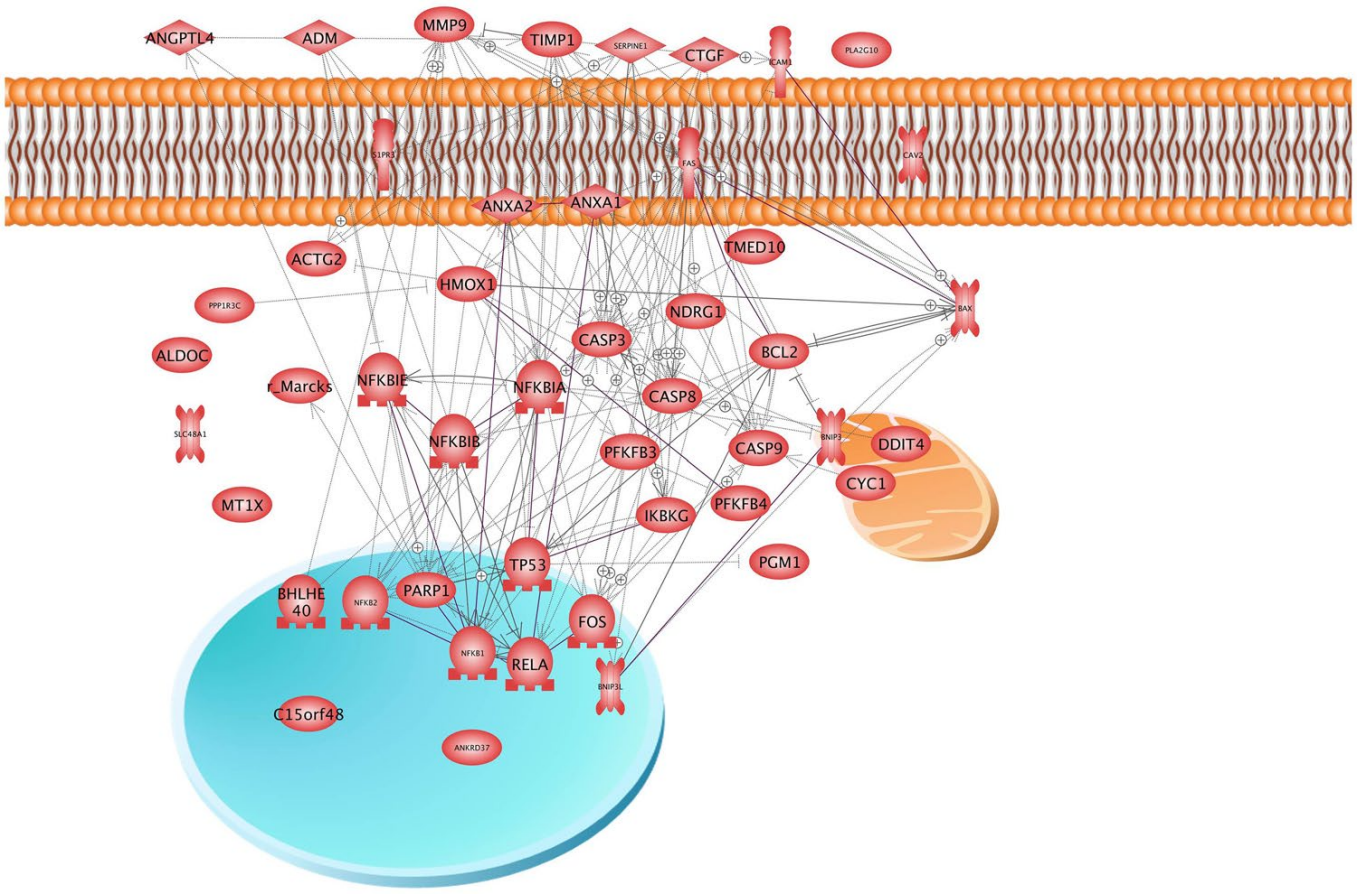
Pathway studio analysis of proteins whose genes were analysed in the study by the gene array analysis and qPCR. Arrows indicate interaction. Mitochondria (yellow), cell membrane (red) and nucleus (blue) are indicated to support localization of the various proteins.

Because 10 of the 19 genes found significantly downregulated are related to oxygen homeostasis, we applied the Pathway Studio analysis to see whether there is an interaction between them (Figs 6 and 7). Interestingly, we did not see significant interactions of these factors, neither at protein nor at gene level. Interaction at gene and protein level was, however, detected, when all 23 factors shown in Table 3 were analyzed. A cross-linking goes through 7 of the 23 genes (Fig. 5B) which code for 3 extracellular, 1 membrane, 5 nuclear, 2 mitochondrial and 12 cytosolic proteins (see also Fig. 7, Table 3). Hemoxygenase (*HMOX1*) is the most networked gene.

In order to examine, whether the genes determined by qPCR as upregulated in MCS cells interact with the differentially expressed genes detected in the gene array analysis, we subjected the experimental results to the Pathway studio analysis. The candidates comprised 41 items including RELA (NFKB3) and Hemox-1. They completed a complicated network consisting of 31 of the 41 factors at gene (Fig. 6) and protein levels (Fig. 7). Figures 5 and 6 indicate that the genes recognized before to be important in spheroid formation and the genes noticed in gene array analysis very well fit into the networks (see also Table 3). *HMOX1* and *RELA* show strong interaction at a genetic level (Fig. 6), but no mutual influence has so far been detected on the protein level (Fig. 7). In addition, the *ICAM1* gene, which codes for a cell adhesion protein is under the positive influence of upregulated genes such as *FAS*, *PARP1*, *P53*, *CTGF*, and *NFKB1*, but may be suppressed by caspase-3. Besides of *CTGF*, these genes are significantly upregulated only in MCS cells.

### Impact of targeting PARP and NFκB on spheroid formation

**Figure 8:**
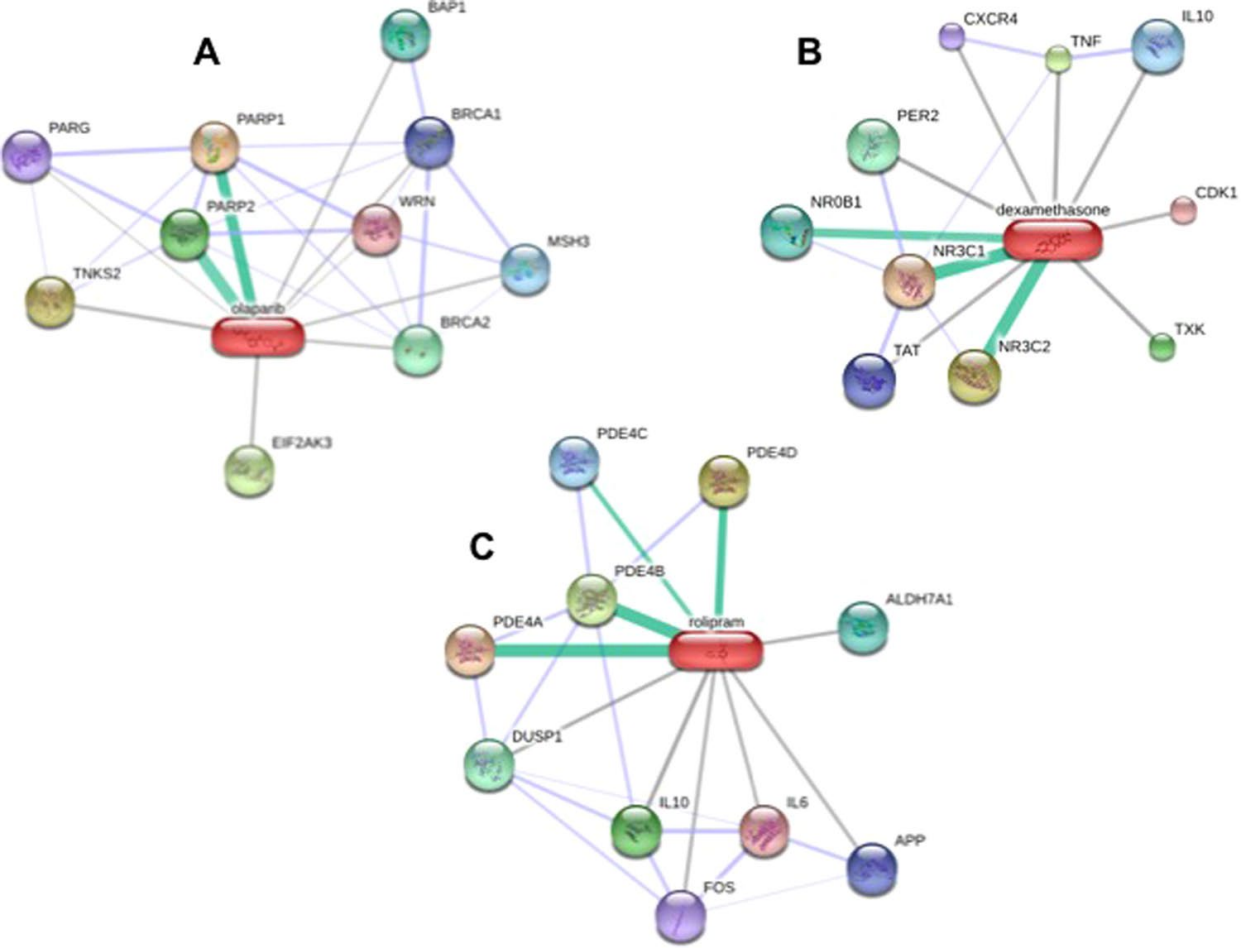
Pharmacological studies. STITCH 4 search for targets of olaparib (**A**), dexamethasone (**B**) and rolipram (**C**). Primary targets are indicated by a green bar between the drug and the protein. Factors affected to a minor degree are shown by grey lines^86^.

In order to evaluate the functional effects of PARP and NFκB, we performed inhibition assessments using the drugs olaparib, dexamethasone (DEX) and rolipram. Figure 8 shows the target proteins of the three drugs. According to a STITCH 4 database search (chemical-protein interaction networks; http://stitch.embl.de/), it can be assumed that DEX preferentially inhibits NROB1, NR3C1 and NR3C2 (Fig. 8, green bars), but has side effects cross-reacting with a couple of other factors including IL10 and CDK1 (Fig. 8). Similarly, olaparib inhibits PARP1 and PARP2 (Fig. 8, green bars) and has additional effects on other eight proteins (Fig. 8). Rolipram mainly blocks the action of PDE4A, PDE4B and PDE4D (Fig. 8, green bars). To some degree it also interacts with factors like IL6, IL10, APP, and FOS.

To determine the formation of spheroids when molecules of interest are inhibited, we treated the MCF-7 cells with various concentrations of olaparib (0 M, 2.5 µM, 5 µM and 10 µM), DEX (0 M, 0.01 µM, 0,1 µM, 1 µM) and rolipram (0 M, 1 µM, 10 µM), while exposed to the RPM for 24 h. To exclude toxic effects of the used solvent and/or the drugs on the MCF-7 cells, we prepared 24 h static 1*g*-experiments with solvent and the mentioned drug concentrations (Supplemental Fig. 1). After a 24 hour-exposure, the cells were stained with acridine orange/ethidium bromide to examine the cell viability (Supplemental Fig. 1). None of the used concentrations of solvent and drugs had a cytotoxic effect on the cells, as presented by the green staining, while no red staining of the nuclei was detectable (Supplemental Fig. 1). In addition, no increased cell detachment or formation of cell aggregates was noticed due to drug or solvent supplementation.

**Figure 9:**
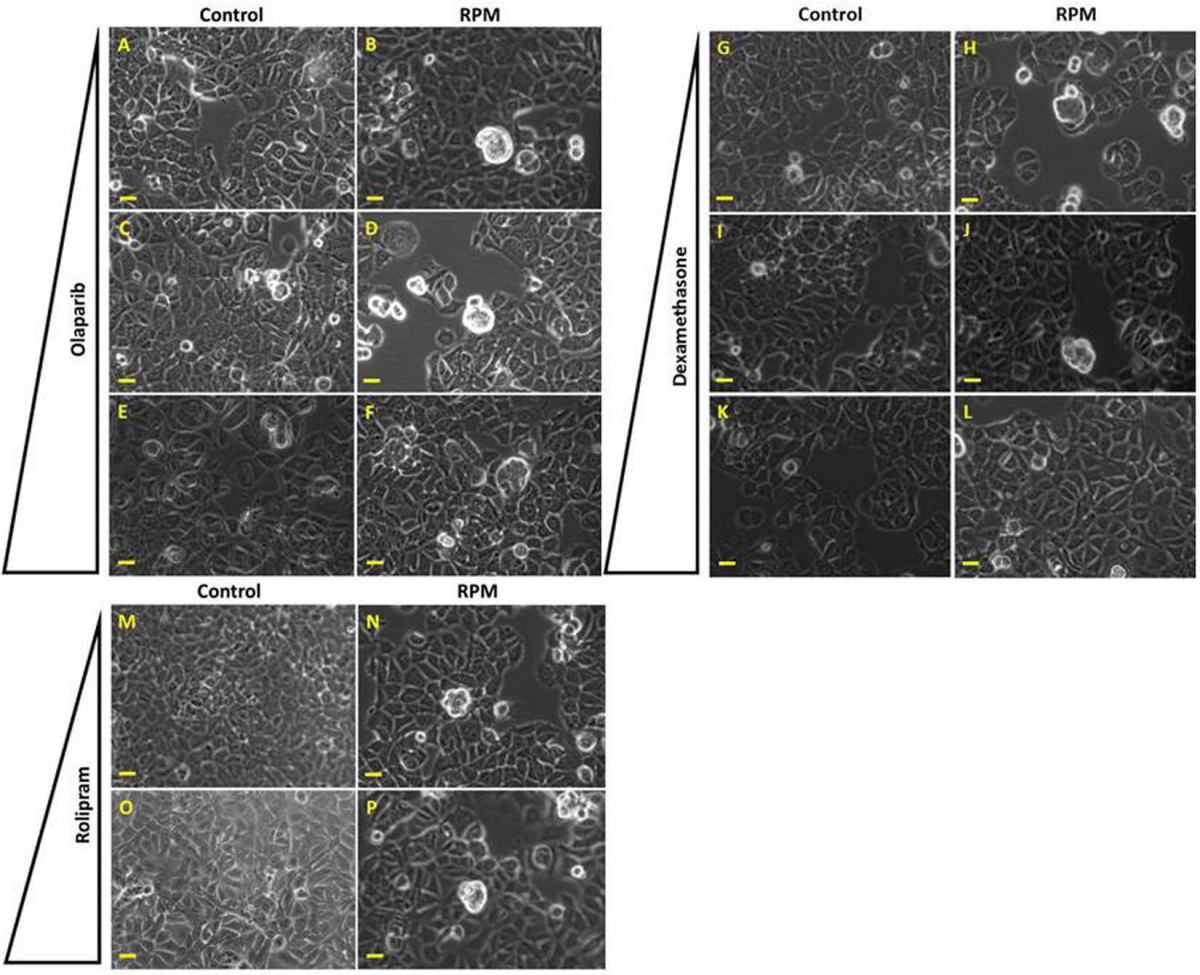
Phase contrast microscopy of drug-treated cells exposed to the RPM. Olaparib-treated cells static control: (**A**) 2.5 µM, (**C**) 5 µM, (**E**) 10 µM. Olaparib-treated cells exposed to the RPM for 24 h: (**B**) 2.5 µM, (**D**) 5 µM, (**F**) 10 µM. DEX-treated cells static control: (**G**) 0.01 µM, (**I**) 0.1 µM, (**K**) 1 µM. DEX-treated cells exposed to the RPM for 24 h: (**H**) 0.01 µM, (**J**) 0.1 µM, (**L**) 1 µM. Rolipram-treated cells static control: M) 1 µM, (**O**) 10 µM. Rolipram-treated cells exposed to the RPM for 24 h: (**N**) 1 µM, (**P**) 10 µM. The pyramid indicates increasing drug concentration. Scale bar: 100 µm.

We exposed the MCF-7 cells to the RPM for 24 h including the drug concentrations mentioned. While treatment with olaparib and rolipram did not show visible effects on the formation of early spheroids (Fig. 9A–F,M–P), the number of visible MCSs seemed to decrease with increasing concentration of DEX (Fig. 9G–L). Acridine orange/ethidium bromide staining revealed viable cells in RPM-AD cells as well as in MCS (Supplemental Fig. 1N, arrow indicates a MCS).

## Discussion

Organisms live on Earth under the permanent influence of gravity. Removing this influence results in remodeling of various tissues in humans. For example bone loss and muscle atrophy can be observed in astronauts and cosmonauts after long-term spaceflights^35^. In addition, various changes in different types of human cells were detected. Examples are macrophages producing less reactive oxygen in microgravity compared to 1 *g*^36^ and human thyroid cancer cells which form 3D cell aggregates, when cultured for a longer time in microgravity^2^. This makes microgravity a valuable environment for studies on a number of cellular characteristics not understood so far^37^. However, long-term removal of gravity achieved by spaceflights is very expensive and seldom performed. With the help of ground-based facilities, which were constructed, to simulate microgravity on Earth, some aspects of annulling gravity can be studied. Such devices, including the RPM, trigger at least a part of the incubated cells to detach from the bottom of a culture flask and to form 3D aggregates like they are observed after spaceflights^1,2^. Even though the RPM produces sheer forces, which are nearly completely absent in real microgravity^38,39^, it is generally accepted that the cause of spheroid formation of cells cultured either on a RPM or in space is the absence of cell sedimentation in both conditions^1,2,40^, as no other cause could be identified until today.

Also MCF-7 cells form spheroids when exposed to the RPM^10^. In contrast to thyroid cancer cells, which only form spheres, the MCF-7 cells form 3D structures that have central holes after a five-day RPM-exposure, which remind of duct structures of normal epithelial breast cells^10^. After a 24 h RPM-exposure dense MCS of MCF-7 cells similar to FTC-133 thyroid cancer spheroids or MCS from normal thyroid cells were visible^14,41^.

The MCF-7 breast cancer cell line is characterized as estrogen-receptor (ER)-positive, and progesterone receptor (PR)-positive. It belongs to the luminal A molecular subtype^42^. MCF-7 cells show characteristics of differentiated breast epithelium and are positive for epithelial markers and negative for mesenchymal markers like vimentin^42^. MCF-7 cells have demonstrated the ability to form MCS under static 1*g*-conditions using non-adherent Petri dishes^43^. These MCS can mature after long-term culture to lumen-containing spheroids^43^.

Normal cells such as endothelial blood vessel cells for example form tubular structures when cultured on a RPM^5^. Human chondrocytes form cartilage pieces when cultured in space and on the Rotating Wall Vessel^44^ or when they were exposed to the RPM^45^.

These observations point to the possibility that the technique of microgravity-dependent spheroid formation could be exploited for tissue engineering purposes in the future^46^, perhaps also in breast reconstruction after surgery. In this study, we investigated the early phase of spheroid formation of MCF-7 cells exposed to simulated microgravity created by a RPM.

MCF-7 cells of a parent population, which grow either as MCS cells or as a monolayer showed similar and different features after a 24 hour-exposure to the RPM. Both states of cellular appearance exhibited an increased expression of *CTGF* and *BAX* and a rather similarly decreased expression of adrenomedullin (*ADM*), aldolase (*ALDOC*), angiopoietin-like 4 (*ANGPTL4*), ankyrin repeat domain 37 (*ANKRD37*), BCL2 interacting protein 3 (*BNIP3*), N-myc downstream regulated 1 (*NDRG1*), whose products are affected by oxygen levels^24–30^. The decrease of *ADM*, *ALDOC*, *ANGPTL4*, *ANKRD37*, *BNIP3*, *NDRG1* may be explained by an increase in oxygen concentration within vented culture flasks observed after 24 h of incubation on the RPM^47^. Such an increase may be the reason for the down-regulation of various genes whose products were changed in response to oxygen levels and regulate glycolysis, as the regulation was very similar in adherent and in MCS cells (Supplemental Table 1). In addition, spheroid formation is observed in closed and not-vented incubation chambers during a spaceflight and during clinorotation (clinostat device), which is another method of preventing cell sedimentation on ground^2,40^. This indicates that different oxygen concentrations are not accountable for spheroid formation. Therefore, an enhanced oxygen concentration together with the down-regulation of hypoxia-inducible factor (HIF)-related genes may be an epiphenomenon, when cells are incubated on the RPM in vented culture flasks. As oxygen enhancement cannot be considered to trigger the switch from a 2D to a 3D growth behavior, we focused on genes which have been differently regulated in AD and MCS cells during the early 24 h of incubation.

Differences in gene expression changes were observed in genes of factors playing a role in apoptosis. These genes were clearly elevated in MCS than in AD samples. They comprise *p53*, *CYC1*, *PARP1*, *FAS*, *Casp8* as well as *ANXA1*^48–50^. These elevated apoptotic factors in MCS are accompanied by factors promoting cell survival. One of these genes is sphingosine-1-phosphate receptor 3 (*S1PR3*), the other one is heme oxygenase-1. They are both up-regulated in MCS as detected by microarray and code for proteins favoring cell survival^33,34^.

*HMOX-1* codes for an enzyme, degrading heme and exhibits together with its degradation products, cytoprotective properties^33^. This enzyme may be localized in various cellular compartments. In caveolae HMOX-1 is inhibited by caveolin-1^51^. In thyroid cells an increased concentration of caveolin-1 prevents spheroid formation^52,53^. Therefore, it may not be excluded that HMOX-1 plays a role in spheroid formation.

According to the literature, both HMOX-1 and NFκB p65 were mutually downregulating their gene expressions^54,55^. In our experimental setting on breast cancer cells grown on the RPM, *HMOX-1* was upregulated, while *NFKB3* remained unregulated. The HMOX-1 upregulation could be due to a positive influence of ADM and PARP-1^56,57^. Despite the *NFKB3* gene expression remained unchanged (Fig. 2F), NFκB p65 protein was accumulated in MCS (Fig. 2G) and enriched within the nucleus. This fact points to an enhanced NFκB p65 translocation activity regulated by NFκBIA and NFκBIB (Figs 6 and 7)^18,58^. Becker-Weimann *et al*.^19^ found that NFκB is a key regulator in the formation of organized spheres in breast cancer cells cultured in 3D matrigels. While organized spheres had a low expression of NFκB p65, unorganized spheres presented an upregulation^19^. This is in concert with our findings that NFκB p65 is enhanced in 24 h adherent and MCS cells as these early spheroids showed a random accumulation of cells (Fig. 1). The random accumulation of MCF7 cells was organized in glandular structures with polarized cells after 5 days of cultivation on the RPM^10^. A deeper investigation of the correlation between cell polarization and NFκB in RPM exposed cells will surely shed further light on the process of spheroid formation under microgravity. Furthermore, NFκB p65 translocation triggers the expression of various proteins including *ICAM1*, which in our experiments was upregulated in MCS (Fig. 3H). Hence, NFκB seems to play a central role in spheroid formation, which suggests that manipulation of NFκB activity by biological or pharmacological agents could influence spheroid formation or related processes^59^. The effect of NFκB p65 is directed towards ICAM1 also by PARP1^60^. CTGF, via mitogen-activated protein kinase and NFκB activation, can induce proinflammatory genes in murine tubuloepithelial cells^61^. Interestingly, the *S1PR3* gene was upregulated in the MCS samples (Table 3). A recent paper demonstrated that sphingosine-1-phosphate increases the expansion of cancer stem cells via S1PR3 by a ligand-independent Notch activation in breast cancer^62^.

It is known that high levels of PARP-1 were associated with a poor prognosis in early breast cancer. *PARP1* overexpression was detectable in various cancer cell lines and was associated with malignant progression^63^. We found a high *PARP1* expression in the spheroids and targeted it by PARP inhibition. Thus, we investigated the impact of olaparib (a competitive PARP-1/2 inhibitor) on the growth of MCF-7 breast cancer cells exposed to the RPM and to 1*g*-conditions (Fig. 9). MCF-7 cells had been already treated with olaparib in earlier studies and three different doses were tested (2.5, 5 and 10 µM)^64^. Recent data suggested that PARP inhibitors might be useful to treat estrogen receptor-positive and estrogen-dependent tumors^65^. Here we could show that targeting PARP1/2 with olaparib did not alter the 3D aggregation of the MCF-7 cells cultured on the RPM, which indicated that PARP seems not to be the main key factor responsible for 3D spheroids formation in simulated microgravity.

In a second step, we used DEX to modulate the NFκB activity. It has been shown that the application of DEX promoted the NFκB transcriptional activity in MCF-7 cells^66^. Khan *et al*. identified that NFκB was also regulated by glucocorticoids and their receptor in MCF-7 breast cancer cells^66^. Here, we applied DEX to evaluate its impact on spheroid formation. After a 24-hour RPM-exposure we detected that 1 µM might inhibit the spheroid formation. This finding supports the hypothesis that NFκB might be involved in spheroid formation on the RPM. It is known that DEX suppresses the *IL8* gene expression in airway epithelial cells^67^. We had shown earlier that the application of IL-8 protein facilitated the formation of MCS in thyroid cancer cell lines using the liquid-overlay technique^68^. Therefore, DEX might interact with *IL8* to reduce MCS formation in this study, which has to be confirmed in the future.

In addition, we tested a second agent known to target NF-κB. Rolipram is a cyclic adenosine monophosphate (cAMP)-specific phosphodiesterase (PDE-4) inhibitor and earlier used to influence MCF-7 cancer cells^69^. The agent has shown to prevent the NFκB binding activity in human chorionic cells^70^. In this experimental setting, we can report that the drug is not influencing 3D spheroid formation in the first 24 h of RPM-exposure (Fig. 9).

Future studies are necessary to clarify the exact mechanisms involved in the process of 3D aggregation and spheroid formation, such as genetic knockouts or knockins in cell lines^71^.

In addition, the genes *CTGF*, *FAS* and *P53*, which were upregulated in MCS and their products, have positive influence on the gene expression of *ICAM1*^72,73^. ICAM1 is a surface protein, mainly detectable in endothelial cells, but also expressed in human breast cancer cells^74^. It may contribute to the cell-cell interaction required for spheroid formation either by direct binding to integrin beta 2^75^ or by changing the structure of the cell adhesion complex as it was observed recently on normal thyroid cells^17^. Interestingly, during earlier studies on MCF-7 cells, we detected a downregulation of *ICAM1* in AD and MCS cells after a 24-hour-exposure to the RPM^10^. The reason might be either due to the changed serum supplementation, as growth factor concentration is unpredictable^76^, or due to the random walk of the RPM which incorporates different variances of stress^39^. In both cases the onset of apoptosis may vary, which reduces *ICAM1* expression via caspase-3^11,77^.

Taken together, our experiments suggest that NFκB family members and *HMOX-1* interact on a gene level, when breast cancer cells transit from a 2D to a 3D growth on the RPM. They are changed in the same direction, when adherent and MCS-cells are compared (Fig. 6). Whether these alterations are accidental parallel events or mutually caused remains to be determined. Whether the up-regulated *ANXA2* or the down-regulated *MARCKS* genes which both code for cytoskeleton interacting proteins or the genes of *CAV2*, *TIMP1*, *PAI1* and *MMP-9*, which either code for membrane proteins or for enzymes regulating the extracellular matrix constitution, contribute to this process remains to be determined in future studies. Interestingly, *CAV2*, *TIMP1*, and *PAI1* show an up-regulation in these experiments, but have exhibited together with *MMP9* a downregulation in earlier experiments on thyroid cells^9^.

In addition, our studies deliver new knowledge about how these cells might behave in real microgravity. This data can be used to prepare future spaceflight missions. Hence, using these and earlier results as a basis^10,78,79^, we plan to conduct a future NASA and DLR space experiment like the successfully flown Cellbox-1 (NanoRacks-CellBox-Thyroid Cancer: http://www.nasa.gov/mission_pages/station/research/experiments/1648.html ^52,53^) onboard the International Space Station in order to increase the current knowledge of the behavior of human breast cancer cells under real microgravity in space with a special focus on early cytoskeletal changes and 3D growth.

## Methods

### Cell culture

MCF-7 human breast adenocarcinoma cells (Fig. 1A) were purchased from the American type culture collection (ATCC) (Manassas, VA, USA). Cells were cultivated in RPMI 1640 medium (Life Technologies, Naerum, Denmark) supplemented with 10% fetal calf serum (FCS) (Biochrom, Berlin, Germany) and 1% penicillin/streptomycin (Biochrom) at 37 °C and 5% CO_2_. One day prior to the experimental run on the RPM, cells were seeded either in slide flasks (Thermo Fisher Scientific, Roskilde, Denmark) (3 × 10^5^ cells/cm²) for fluorescence staining or in T25 (1 × 10^6^ cells) vented cell culture flasks (Sarstedt, Nümbrecht, Germany) for RNA and protein extraction. Before starting the run, flasks were filled up with medium, taking care that no air bubbles remain. A detailed procedure has been published previously^9,23^. To test the viability of the cells in the multicellular spheroids (MCS), the MCS were collected after 24 h (Fig. 1B) and seeded in slide flasks. The adhesion and migration behavior of the cells of the MCS was examined by phase contrast microscopy after 2 h, 4 h and 24 h (Fig. 1C–E).

### Drug treatment

For targeting molecules of interest, we seeded 10^6^ MCF-7 cells in slideflasks. After 24 h the cells were synchronized for 4 h and afterwards treated with the respective chemical agents for 24 h during RPM-exposure or without RPM-exposure. To target PARP we used the PARP 1/2 inhibitor olaparib (Selleckchem, Absource Diagnostics, Munich, Germany). We prepared a stock solution in DMSO. The aliquots were stored at −80 °C until use. Concentrations of 2.5, 5, or 10 µM olaparib in medium were applied^64^. Negative controls were incubated with DMSO only.

For targeting NFκB we applied dexamethasone (DEX) (Sigma-Aldrich, Taufkirchen, Germany). According to Bruxant *et al*.^80^ and Khan *et al*.^66^ we treated the MCF-7 cells with DEX (0 M, 0,001 µM, 0,1 µM, 1 µM). The MCF-7 cells were treated with DEX dissolved in water for 24 h.

Moreover, we applied the PDE4 inhibitor rolipram. We used the following doses 0 M, 1 µM, and 10 µM^69^. Rolipram was first prepared as a stock solution in ethanol. Control MCF-7 cells were treated with an equivalent volume of the solvent.

### Random Positioning Machine

The desktop RPM (Airbus Defense and Space (ADS), Leiden, The Netherlands) was located in a standard incubator with 37 °C and 5% CO_2_. The RPM was operated in real random mode with random direction and interval and a maximum speed of 12.5 revolutions per minute. Sample flasks to be tested were placed onto the middle frame with a maximal distance of 7 cm to the center of rotation allowing a µ*g* quality between 10^−4^ and 10^−2^ *g*, which is reached over time^40,81^. Corresponding static 1*g*-controls, which were completely filled with medium, were placed next to the RPM in the same incubator (n = 15 samples each group/run).

### Phase contrast microscopy

Cells were observed and photographed using an Axiovert 25 Microscope (Carl Zeiss Microscopy, LLC, USA) and a Canon EOS 550D camera (Canon GmbH, Krefeld, Germany)^23^.

### Sample collection

After 24 h the cells were investigated and photographed. The MCS were collected by mild centrifugation at 3000 *g* for 5 min and 4 °C and stored in liquid nitrogen. Harvesting of the adherent cells was performed, by using a cell scraper after carefully adding ice-cold phosphate buffered saline (PBS, Life Technologies). The suspension was collected and centrifuged at 3000 *g* for 5 min and 4 °C followed by discarding the PBS and storage of the pellet in liquid nitrogen.

### Acridine orange/ethidium bromide staining

Control and RPM-exposed MCF-7 cells of both phenotypes MCS and adherently growing cells were stained with acridine orange/ethidium bromide (Molecular Probes, Darmstadt, Germany) as performed in previous studies^82^. The stained MCF-7 cells were immediately investigated by using a Zeiss LSM 710 confocal laser scanning microscope (Zeiss, Jena, Germany) as previously described^9^.

### Indirect immunofluorescence staining of NFκB

Immunofluorescence analysis of NFκB p65 was performed on 80% confluent MCF-7 cells after a 24 hour exposure to the RPM. The cells were fixed with 4% paraformaldehyde for 25 minutes at room temperature (RT), permeabilized with 0.25% Triton™ X-100 for 10 minutes, and blocked with 5% BSA for 1 h at RT. Afterwards, the cells were labelled with NFκB [p65] rabbit polyclonal antibody (Thermo Fisher Scientific) at 2 µg/mL in 1% BSA and incubated overnight at 4 °C, then labelled with Alexa Fluor 488 goat anti-rabbit IgG secondary antibody (Thermo Fisher Scientific) at a dilution of 1:400 for 1 h at RT and washed 3 times. For nuclear staining, we used DAPI (4′,6-diamidin-2-phenylindol) (Sigma-Aldrich, Taufkirchen, Germany) for 5 min and washed the cells twice with DPBS. For evaluation, the slides were mounted with Fluoroshield (Sigma-Aldrich, Taufkirchen, Germany) and analysed with a Zeiss LSM 710 confocal laser scanning microscope^9^.

### Western Blot Analysis

Western blot analysis was performed as recently published^17^. The RPM experiment for the Western blot analyses was performed three times. In each of these experiments five different culture flasks were mounted on the RPM. In parallel five 1*g*-control flasks were cultured next to the RPM. At the end of the experiment cells were harvested and an aliquot from each flask was subjected to Western blot analysis. In RPM-samples we detected two phenotypes (RPM-AD cells and RPM-MCS). Hence, the Western blot contains ten lanes loaded with RPM samples (AD and MCS), and five lanes loaded with 1*g*-control samples. The Western blot experiment was repeated thrice. The concentration was adjusted to a total protein load of 30 µg per well in Laemmli buffer. Criterion XT 4–12% precast gels (Biorad, Hercules, USA) were loaded and run for 1 h at 150 volts. Transfer to a PVDF membrane was performed at 100 volts and 30 minutes. Membranes were blocked in 0.3% I-Block (Applied Biosystems, Foster City, USA) in TBS-T overnight. The antibodies listed in Table 1 were applied for 2 h at room temperature followed by washing steps. The applied secondary antibody, a Horseradish peroxidase (HRP)-linked antibody was utilized at a dilution of 1:4000 (Cell Signaling Technology, Inc., Danvers, MA, USA) for additional 2 h at room temperature. Membranes were developed using Biorad Clarity Western ECL and imaged with an Image Quant LAS 4000 mini (GE Healthcare Life Science, Freiburg, Germany). Cofilin (CFL1) [EPR6375] (ABCAM, Cambridge, UK) was used as a loading control. The membranes were analyzed using ImageJ software (U.S. National Institutes of Health, Bethesda, MD, USA; http://rsb.info.nih.gov/ij/) for densitometric quantification of the bands^83^.

### RNA and protein extraction

The RNA and protein extraction were performed using the AllPrep RNA/Protein kit (Qiagen GmbH, Hilden, Germany) according to the manufacturer’s protocol. The concentrations were determined with the spectrophotometer Ultrospec 2100 pro (Amersham Biosciences, Amersham, Great Britain).

### Quantitative real-time PCR

Complementary DNA was produced using the First Strand cDNA Synthesis Kit (Thermo Fisher Scientific) following manufacturer’s instructions. qrtPCR was performed using the SYBR^®^ Select Master Mix (Applied Biosystems, Darmstadt, Germany) and the 7500 Real-Time PCR System (Applied Biosystems) to determine the expression levels of target genes, shown in Table 2. Selective primers were designed to span exon-exon boundaries and to have a Tm of 60 °C using Primer Express software (Applied Biosystems), and were synthesized by TIB Molbiol (Berlin, Germany). Samples were measured in triplicate and were normalized to the housekeeper 18 S rRNA. Comparative threshold cycle (ΔΔCT) methods were used for relative quantification of transcription levels, with 1 *g* set as 100%^84^.

### Microarray technique

The 25 Illumina HumanWG-6_V2_0_R3 arrays have been normalized using the BeadStudio Gene Expression Module v3.3.7, and quantile normalization without background correction. After quantile normalization and exclusion of low or not expressed genes (minimum Illumina detection p-value > 0.05) a parametric ANOVA comparing the conditions control, AD and MCS was performed. Probes which undergo 5% FDR^85^ were selected as differential expressed. Differentiation of the expression profiles was performed using hierarchical and k-mean clustering. The cluster analysis was done using Partek Genomic Suite 6.3 applying hierarchical average linkage clustering and k-mean clustering with Euclidean distance function on standardized log2 signal values. K was selected by analyses of the hierarchical clustering dendrograms.

### Pathway analyses

To investigate mutual regulation of genes and to visualize localization and interactions between proteins, we entered relevant UniProtKB entry numbers in the Pathway Studio v.11 software (Elsevier Research Solutions, Amsterdam, The Netherlands). Graphs were generated for gene expression and protein regulation and binding. The method was described previously^9,52^. STITCH 4 (Chemical-Protein Interaction Networks, European Molecular Biology Laboratory (EMBL), Heidelberg, Germany) was applied to investigate the interaction of DEX, olaparib and rolipram with their targets. The data is given in Fig. 8.

### Statistical evaluation

Statistical evaluation was performed using SPSS 15.0 (SPSS, Inc., Chicago, IL, USA). The Mann-Whitney-U-Test was used to compare 1 *g* and s-μ*g* conditions, as well as AD cells and MCS cells. All data is presented as mean ± standard deviation (SD) with a significance level of *p < 0.05.

## Electronic supplementary material

Supplemental Table 1 and Supplemental Fig. 1
