# Supplementary material for: The role of NFκB in spheroid formation of human breast cancer cells cultured on the Random Positioning Machine
Source: Sci Rep. 2018 Jan 17;8:921. doi: 10.1038/s41598-017-18556-8 (PMC5772637; doi:10.1038/s41598-017-18556-8)
Supplement: Supplementary file 1 — Supplemental Table 1 and Supplemental Fig. 1 [file 41598_2017_18556_MOESM1_ESM.pdf]

# The role of NFκB in spheroid formation of human breast cancer cells cultured on the Random Positioning Machine

Sascha Kopp[1], Jayashree Sahana[2], Tawhidul Islam[2], Asbjørn Graver Petersen[2], Johann Bauer[3], Thomas J. Corydon[2,4], Herbert Schulz[5], Kathrin Saar[6], Norbert Huebner[6], Lasse Slumstrup[2], Stefan Riwaldt[2], Markus Wehland[1], Manfred Infanger[1], Ronald Luetzenberg[1] and Daniela Grimm[1,2*]

[1] Clinic for Plastic, Aesthetic and Hand Surgery, Otto-von-Guericke-University Magdeburg, D-39120 Magdeburg, Germany

[2] Department of Biomedicine, Aarhus University, Wilhelm Meyers Allé 4, DK-8000 Aarhus C, Denmark

[3] Max-Planck Institute of Biochemistry, D-82152 Martinsried, Germany

[4] Department of Clinical Medicine, Aarhus University, Aarhus, Denmark

[5] Cologne Center for Genomics, University of Cologne, D-50931 Cologne, Germany

[6] Max-Delbruck-Center for Molecular Medicine, D-13092 Berlin-Buch, Germany

**Supplemental Table 1: Genes and open reading frames detected by microarray analyses regulated by at least 1.4-fold in AD and/or MCS compared to control**

| Cluster | Symbol | AD vs. CON | MCS vs. CON | MCS vs. AD |
|---|---|---|---|---|
| Genes of Cluster 1: RPM upregulated | *ADM* | -3.97341 | -3.7179 | 1.06872 |
| | *ADORA2B* | -1.30814 | -1.52168 | -1.16323 |
| | *AK3L1* | -1.70247 | -1.75028 | -1.02808 |
| | *ALDOC* | -2.72112 | -2.93859 | -1.07992 |
| | *ANG* | -1.67669 | -1.98727 | -1.18524 |
| | *ANGPTL4* | -2.82266 | -2.52424 | 1.11822 |
| | *ANKRD37* | -3.30216 | -3.47036 | -1.05094 |
| | *B3GNT4* | -1.26283 | -1.40584 | -1.11324 |
| | *BHLHB2* | -2.6305 | -2.12209 | 1.23958 |
| | *BIK* | -1.39295 | -1.41051 | -1.01261 |
| | *BNIP3* | -2.06507 | -1.99325 | 1.03603 |
| | *BNIP3L* | -2.10573 | -2.65328 | -1.26003 |
| | *C12orf24* | -1.46439 | -1.64136 | -1.12085 |
| | *C17orf58* | -1.27881 | -1.49989 | -1.17287 |
| | *C18orf19* | -1.29094 | -1.57219 | -1.21786 |
| | *C1orf51* | -1.9015 | -1.78685 | 1.06417 |
| | *CA9* | -1.52825 | -1.59152 | -1.0414 |
| | *CCNG2* | -1.62608 | -1.51019 | 1.07674 |
| | *CITED2* | -1.54627 | -1.77648 | -1.14888 |
| | *DDIT4* | -2.36264 | -2.80513 | -1.18729 |
| | *DHX40* | -1.41572 | -1.48235 | -1.04706 |
| | *EGLN1* | -1.38666 | -1.44326 | -1.04082 |
| | *ERRFI1* | -1.82971 | -1.32601 | 1.37986 |
| | *FAM117B* | -1.34774 | -1.50396 | -1.11591 |
| | *FOS* | -2.23903 | -1.6201 | 1.38203 |
| | *HBP1* | -1.30694 | -1.40915 | -1.07821 |
| | *HK2* | -1.68224 | -1.27388 | 1.32057 |
| | *ILVBL* | -1.27771 | -1.62488 | -1.27171 |
| | *INSIG2* | -1.63554 | -1.69319 | -1.03524 |
| | *ITGB2* | -1.22775 | -1.43314 | -1.16729 |
| | *JAG2* | -1.49104 | -1.49822 | -1.00482 |
| | *JMJD1A* | -1.47657 | -1.47307 | 1.00238 |
| | *LMTK3* | -1.21146 | -1.45332 | -1.19965 |
| | *LOC100131785* | -1.27989 | -1.48939 | -1.16369 |
| | *LOC100132377* | -1.44876 | -1.4184 | 1.0214 |
| | *MMEL1* | -1.5038 | -1.95736 | -1.30161 |
| | *MT1A* | -1.52935 | -1.81461 | -1.18652 |
| | *MT1X* | -2.30906 | -2.49814 | -1.08189 |
| | *MT2A* | -1.4035 | -1.86267 | -1.32717 |
| | *NDRG1* | -2.95903 | -2.78486 | 1.06254 |
| | *NUDT7* | -1.22442 | -1.45485 | -1.1882 |
| | *P4HA1* | -1.47165 | -1.68115 | -1.14236 |
| | *P4HA2* | -1.5836 | -1.7482 | -1.10394 |
| | *PFKFB3* | -2.10202 | -3.13231 | -1.49014 |

| | | | | |
|---|---|---|---|---|
| | *PFKFB4* | -3.20572 | -2.84829 | 1.12549 |
| | *PGM1* | -1.77618 | -2.05219 | -1.15539 |
| | *PLIN2* | -1.47455 | -1.52893 | -1.03687 |
| | *PPP1R3C* | -1.72085 | -2.07604 | -1.20641 |
| | *RNASE4* | -1.46108 | -1.56088 | -1.06831 |
| | *RRAGD* | -1.77969 | -1.39958 | 1.27159 |
| | *SPAG4* | -1.6619 | -1.70641 | -1.02678 |
| | *SPRY1* | -1.50611 | -1.5772 | -1.0472 |
| | *STC1* | -1.60782 | -1.8989 | -1.18104 |
| | *TMEM159* | -1.22318 | -1.40019 | -1.14472 |
| | *VEGFB* | -1.35306 | -1.52893 | -1.12998 |
| | *VLDLR* | -1.61771 | -1.912 | -1.18192 |
| | *WSB1* | -1.91009 | -1.44352 | 1.32322 |
| Genes of Cluster 2: Control upregulated. MCS downregulated | *ACTG2* | 1.67742 | 2.17152 | 1.29456 |
| | *AKR1C2* | 1.17607 | 1.7109 | 1.45476 |
| | *ALDH3A1* | 1.12885 | 1.50532 | 1.3335 |
| | *DGKD* | 1.1551 | 1.5032 | 1.30136 |
| | *ETF1* | 1.18183 | 1.4804 | 1.25264 |
| | *EXOSC8* | 1.32935 | 1.4463 | 1.08798 |
| | *FLJ20489* | 1.31299 | 2.07465 | 1.58009 |
| | *G6PD* | 1.13154 | 1.59176 | 1.40672 |
| | *GAB2* | 1.10554 | 1.42793 | 1.29162 |
| | *HMOX1* | 1.23716 | 2.78409 | 2.25039 |
| | *LPPR2* | 1.35202 | 1.45429 | 1.07565 |
| | *MCM4* | 1.29106 | 1.79161 | 1.38771 |
| | *NAP1L4* | 1.19352 | 1.4509 | 1.21564 |
| | *PPP2R2C* | 1.17399 | 1.56507 | 1.33312 |
| | *RAB15* | 1.12956 | 1.64821 | 1.45916 |
| | *RAB5C* | 1.28289 | 1.68525 | 1.31363 |
| | *RPL35* | 1.34152 | 1.46439 | 1.09159 |
| | *S1PR3* | 1.46902 | 2.07908 | 1.41529 |
| | *STIP1* | 1.15557 | 1.46048 | 1.26386 |
| | *STX3* | 1.20952 | 1.61906 | 1.33859 |
| | *TBC1D14* | 1.11255 | 1.43264 | 1.28771 |
| | *TXNRD1* | 1.24829 | 1.83333 | 1.46867 |
| Genes of Cluster 3: MCS upregulated | *ACOT2* | -1.04912 | -1.40627 | -1.34043 |
| | *APPBP2* | -1.0582 | -1.53284 | -1.44854 |
| | *C15orf48* | -1.28973 | -2.14925 | -1.66644 |
| | *CLDN7* | 1.00836 | -1.65837 | -1.67223 |
| | *CTSH* | -1.0351 | -1.53903 | -1.48684 |
| | *DHX40* | -1.2358 | -1.73101 | -1.40072 |
| | *GGH* | -1.04035 | -1.65328 | -1.58916 |
| | *GLB1* | -1.08471 | -1.46916 | -1.35443 |
| | *GNG12* | -1.08525 | -1.42611 | -1.31408 |
| | *GPR137B* | -1.06179 | -1.50145 | -1.41408 |
| | *GPR160* | -1.11668 | -1.46794 | -1.31456 |
| | *GRN* | -1.17096 | -1.43715 | -1.22732 |
| | *HNMT* | -1.169 | -1.53818 | -1.3158 |
| | *IFITM2* | 1.03544 | -1.43876 | -1.48975 |
| | *IL27RA* | 1.03708 | -1.49168 | -1.54699 |

| | | | | |
|---|---|---|---|---|
| | *LFNG* | -1.04554 | -1.46089 | -1.39726 |
| | *LIPA* | -1.04431 | -1.86092 | -1.78197 |
| | *LOC650515* | -1.01805 | -1.42241 | -1.3972 |
| | *LOC732007* | -1.33617 | -2.60017 | -1.94599 |
| | *MARCKS* | -1.26029 | -1.84646 | -1.46511 |
| | *MCM7* | -1.23023 | -2.21407 | -1.79972 |
| | *MDK* | -1.04404 | -1.69706 | -1.62548 |
| | *MSMB* | 1.1377 | -1.95969 | -2.22955 |
| | *MYC* | -1.12972 | -1.52496 | -1.34985 |
| | *PLA2G10* | -1.17541 | -2.21319 | -1.8829 |
| | *PMP22* | 1.0385 | -1.43812 | -1.49349 |
| | *PQLC3* | -1.03636 | -1.45337 | -1.40238 |
| | *PRIM1* | -1.06411 | -1.77164 | -1.6649 |
| | *PRIM2A* | -1.0914 | -1.42062 | -1.30165 |
| | *PXMP3* | -1.01232 | -1.60967 | -1.59008 |
| | *SERPINA3* | -1.09919 | -1.72366 | -1.56812 |
| | *SLC38A1* | -1.18081 | -1.5373 | -1.3019 |
| | *SORD* | -1.11082 | -1.41049 | -1.26977 |
| | *SPCS3* | 1.11231 | -1.30143 | -1.44759 |
| | *TCEAL1* | -1.10651 | -1.4946 | -1.35074 |
| | *TFF3* | -1.03027 | -1.60035 | -1.55333 |
| | *TMED10* | -1.13444 | -2.60338 | -2.29484 |
| | *TMEM59* | -1.07146 | -1.45436 | -1.35736 |
| | *TOM1L1* | -1.0269 | -1.46504 | -1.42666 |
| | *UBE1DC1* | -1.06943 | -1.77201 | -1.65697 |
| Genes of Cluster 4: MCS downregulated | *C6orf62* | -1.00139 | 1.41788 | 1.41984 |
| | *CCDC34* | -1.30124 | 1.10977 | 1.44408 |
| | *COBLL1* | -1.01386 | 1.42085 | 1.44055 |
| | *CORO1C* | 1.05372 | 1.57059 | 1.49052 |
| | *FTH1* | 1.02728 | 1.40674 | 1.36938 |
| | *GPX2* | 1.07206 | 1.67852 | 1.5657 |
| | *KIAA1370* | -1.09305 | 1.45512 | 1.59053 |
| | *LOC648526* | -1.1167 | 1.25892 | 1.40584 |
| | *LRRFIP2* | 1.05485 | 1.51037 | 1.43183 |
| | *OKL38* | 1.07716 | 1.47737 | 1.37154 |
| | *PALLD* | 1.04059 | 1.52617 | 1.46665 |
| | *PANX2* | 1.03117 | 1.4836 | 1.43875 |
| | *PPP1R15A* | -1.30598 | 1.18516 | 1.54779 |
| | *PSME4* | 1.03028 | 1.52236 | 1.47761 |
| | *PYGO2* | -1.05488 | 1.34032 | 1.41388 |
| | *RAB21* | 1.04406 | 1.51264 | 1.44881 |
| | *RN7SK* | -1.06423 | 1.48099 | 1.57612 |
| | *SRF* | -1.10582 | 1.36646 | 1.51106 |
| | *TNPO1* | 1.01483 | 1.47922 | 1.45761 |
| | *TNPO3* | -1.02202 | 1.60356 | 1.63886 |
| | *UNKL* | 1.00753 | 1.651 | 1.63866 |
| | *WDR82* | -1.07464 | 1.33563 | 1.43532 |

**Supplementary Information**

**Supplemental Figure 1:** Acridine orange/ethidium bromide staining revealed a green fluorescence in all cells after 24 h, which indicates viability.

A-M: 1*g*-conditions with solvent only (A-F) and with drugs (G-M) (insert: positive control (PC) of acridine orange/ethidium bromide assay after 5 min of incubation). N: exemplary viable cells in the MCS after 0.01 µM DEX-treatment after a 24-hour RPM-exposure (insert: PC of dead cells). The arrow shows one single MCS per visual field. Scale Bar: 100 µm

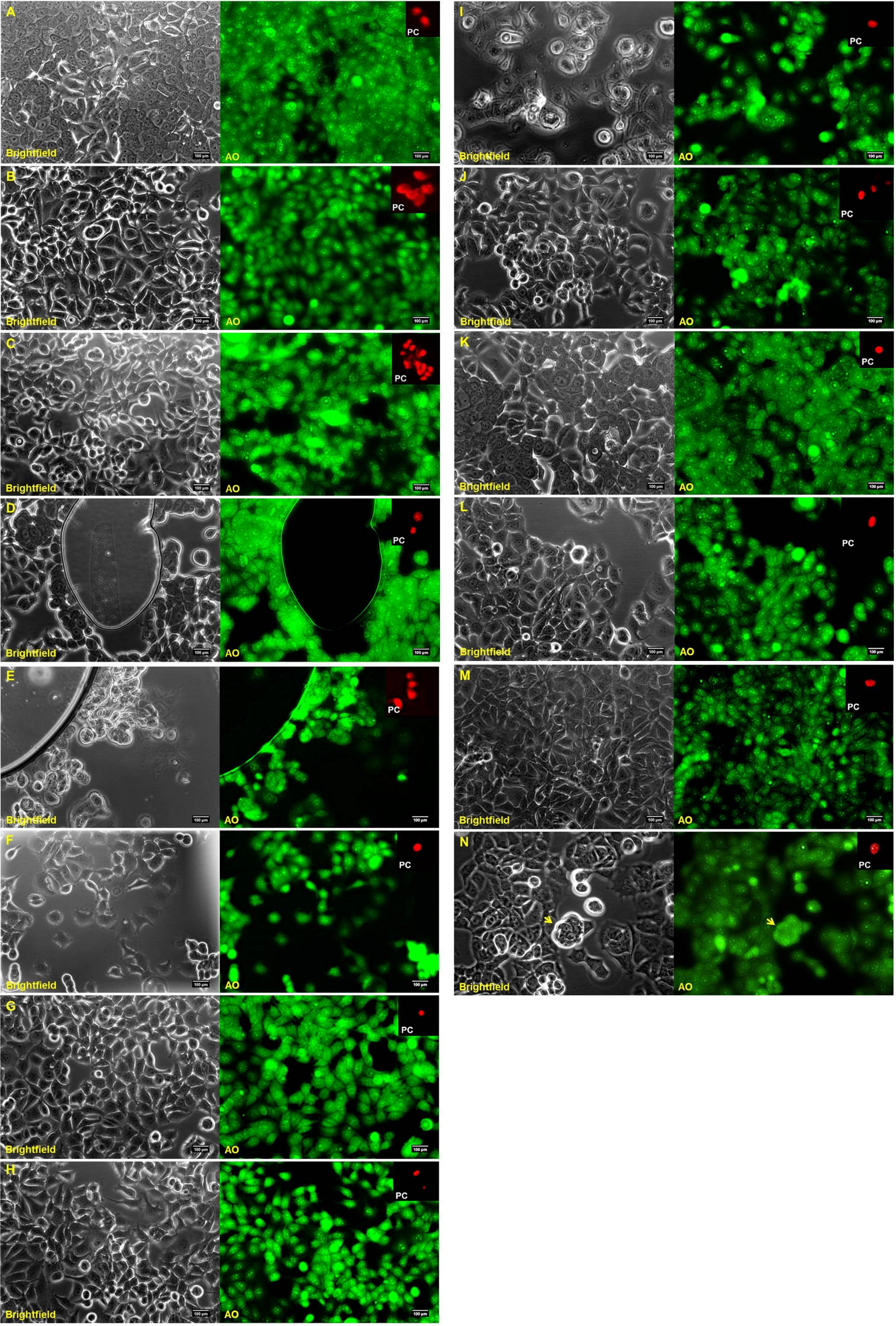
A
Brightfield
AO
PC
B
C
D
E
F
G
H
I
J
K
L
M
N
100 µm
